# Supplementary material for: Inherited Proteoglycan Biosynthesis Defects—Current Laboratory Tools and Bikunin as a Promising Blood Biomarker
Source: Genes (Basel). 2021 Oct 20;12(11):1654. doi: 10.3390/genes12111654 (PMC8625474; doi:10.3390/genes12111654)
Supplement: Supplementary file 1 [file genes-12-01654-s001.zip › genes-1418070-supplementary.pdf]

Supplementary Table S1. Overview of some proteoglycans and their pathophysiological involvements.

Location	Designation	Type of GAG chain	Role
Intracellular	Serglycin (SG)	Heparin/CS	Formation of intracellular storage granules in mast cells [1].
Cell surface	Syndecans	HS/CS	Regulation of vascular development and wound healing [2].
	Glypicans	HS	Regulation of Wnt and FGF signaling pathways during growth, development and cancer [3].
Extracellular matrices	Aggrecan	CS/KS	Provides the mechanical properties of cartilage [4].
	Fibromodulin	KS	Regulation of collagen fibrillogenesis, muscle cell growth, cellular reprogramming, and angiogenesis [5].
	Decorin	DS	Promotes collagen fibrillogenesis [6] and inhibits tyrosine kinase receptors [7].
	Biglycan	CS/DS	Promotes collagen fibrillogenesis [8] and promotes osteoblast differentiation and bone maturation [9].
	Collagen XVIII	HS	Structural component of the basement membranes of vascular and epithelial cells. Inhibits angiogenesis and Wnt signalling [10].

Blood	Endocan	DS	Regulation of cell adhesion, migration, proliferation, and angiogenesis in cancer and wound healing [11].
	Inter-α-trypsin inhibitor family	CS	Stabilization of extracellular matrices, anti-plasmin activity, inhibition of complement activation and protection against kidney stone formation [12].
	PG-100	CS	Inhibition of atherosclerosis formation by trapping of low-density lipoprotein [13]. Growth factor activity triggering differentiation of bone marrow cells [14].
	Apolipoprotein-O	CS	Regulation of fatty acid metabolism and oxidative stress during inflammation [15-16]

Supplementary Table S2. Proteoglycan inherited metabolic diseases: Classification and clinical aspects.

**PG defect subgroup**	**Mutated gene**	**Associated disorder and clinical features**
**Defects of the tetrasaccharide linkage region** (Linkeropathies)	*XYLT1*<br>Initiating xylose transfer to the core protein (Xylβ1-*O*-Ser).	**- Desbuquois dysplasia type 2** (MIM - 615777):<br>Short long bones, joint laxity and dislocations, advanced carpal/tarsal ossification, chondrodysplasia, growth retardation [17].
	XYLT2<br>Initiating xylose transfer to the core protein (Xylβ1-*O*-Ser).	**- Spondyloocular syndrome** (MIM - 605822): Osteopenia/osteoporosis, cataract, deafness, heart defects [18].
	B4GALT7<br>Transfer of the first galactose of the	**- Ehlers-Danlos syndrome (EDS) spondylodysplastic type 1** (MIM - 130070):

	linkage (Galβ1–3Galβ1–4Xylβ1-*O*-Ser).	Joint laxity, hyperelastic skin, short stature, hypotonia, poor wound healing [19].
	B3GALT6<br>Transfer of the second galactose of the linkage region (Galβ1–3Galβ1–4Xylβ1-*O*-Ser).	**- EDS progeroid type 2** (MIM – 615349) and **Spondyloepymetaphyseal dysplasia** (MIM – 271640):<br>Recurrent bone fractures, epimetaphyseal dysplasia, joint laxity and dislocations, cutaneous hyperlaxity, scoliosis, intellectual disabilities, poor wound healing, hypotonia [20].
	B3GAT3<br>Transfer of the terminating glucuronic acid of the linkage region (GlcAβ1–3Galβ1–3Galβ1–4Xylβ1-*O*-Ser).	**Larsen-like syndrome** (MIM – 245600):<br>Joint laxity and dislocations, scoliosis, cutis laxa, heart defects [21].
	FAM20B<br>Phosphorylation of the xylose after the first galactose addition (Gal-Xyl(2-*O*-phosphate)-*O*-Ser).	**- Desbuquois dysplasia** (MIM - 615777) with lethal neonatal short limb dysplasia [22].
**GAG elongation defects**	*CSGALNACT1*<br>First GalNAc transfer to the linkage region: initiation of CS elongation.	**Mild skeletal dysplasia with advanced bone age** (unreferenced):<br>Skeletal dysplasia, short stature, advanced bone age, facial dysmorphism, joint laxity, monkey wrench appearance of the femur, Bell's plasy, neuropathies [23].
	CHSY1<br>[GlcA-GalNAc] polymerization: CS GAG elongation	**- Temtamy preaxial brachydactyly syndrome** (MIM - 605282):<br>Characteristic digits shortening, facial dysmorphism, deafness, growth and mental retardation [24].
	EXTL3<br>First GlcNAc transfer to the linkage region: Initiation of HS elongation.	**- Neuro-immuno-skeletal dysplasia syndrome** (MIM - 617425):<br>Severe immunodeficiency associated to skeletal dysplasia, developmental delay and intellectual disability [25].

	EXT1/EXT2<br>[GlcA-GlcNAc] polymerization: HS GAG elongation	**- Hereditary multiple exostosis syndrome** (MIM - 133700/133701):<br>Benign osteoarticular tumors called exostosis or osteochondromas leading to skeletal dysmorphisms [26-27].
	DSE<br>GlcA epimerization to IdoA: CS conversion to DS	**- EDS musculocontractural type 2** (MIM - 615539):<br>Characteristic craniofacial deformities (large fontanel, hypertelorism, blue sclerae…), skin hyperextensibility, muscle hypoplasia and hypotonia, bruising [28].
**GAG sulfation defects**	*SLC26A2*<br>Cell-surface inorganic sulfate transporter	**- Achondrogenesis type 1B** (MIM - 600972): Micromelia, flat face, short trunk, fetal/perinatal lethality. [29]<br><br>**- Atelosteogenesis type 2** (MIM - 256050): Limb shortening, cleft palate, characteristic facial dysmorphisms, 'hitchhiker' thumbs, possible fetal/perinatal lethality.<br><br>**- Achondrogenesis type 1B** (MIM - 600972): Micromelia, flat face, short trunk, fetal/perinatal lethality.<br><br>**- Atelosteogenesis type 2** (MIM - 256050): Limb shortening, cleft palate, characteristic facial dysmorphisms, 'hitchhiker' thumbs, possible fetal/perinatal lethality.<br><br>**- Diastrophic dysplasia** (MIM - 222600): Short and thick long bones, joint dysplasia, scoliosis, 'hitchhiker' thumbs, club feet, cleft palate, cysts on the external ear.<br><br>**- Recessive multiple epiphyseal dysplasia type 4** (MIM - 226900):<br>Flat epiphyses, hips arthritis, double layered-patella, clubfoot, brachydactyly, joint pain.

CHST3<br>Sulfation of GalNAc residues in the CS chains	**- Spondyloepiphyseal dysplasia with joint laxity (SEDJL) CHST3 type** (MIM - 143095): Kyphosis/scoliosis, Club feet, knees and hips dislocations, short stature [30].
CHST6<br>Sulfation of GlcNAc residues in KS chains	**- Macular corneal dystrophy** (MIM - 217800): Corneal opacity and thinning, photophobia, tearing, progressive vision loss [31].
CHST12, CHST13**<br>Sulfation of GalNAc residues on the CS chain<br>*UST**<br>Sulfation of GlcA/IdoA residue in CS/DS chains	**- Kashin-Beck disease***<br>Joint pain, deformities and limited joint mobility in fingers, shoulders, ankles, knees, wrists, toes. Bone damage and short stature [32].<br>**Unestablished causality*
CHST14 (D4ST1) Sulfation of GalNAc residues on the DS chains	**- EDS musculocontractural type 1** (MIM - 601776):<br>Muscle hypotonia, facial dysmorphisms, joint dislocation, skin hyperlaxity, club feet, bruising [33-34] .
PAPSS2<br>Synthesis of sulfate donors (PAPS)	**- Spondyloepimetaphyseal dysplasia Pakistani type** (MIM - 612847):<br>Short stature, scoliosis, osteoarthritis, mild brachydactyly [35].<br>**- Brachyolmia** (MIM - 271530): Short stature, short trunk, platyspondyly, vertebral deformities,, calcification of costal cartilage, and hormonal disturbances [36-37].

	IMPAD1 (inositol monophosphatase domain-containig 1)<br>Hydrolysis of PAP released from sulfation reactions	**- Chondrodysplasia with joint dislocations, gPAPP type** (MIM - 614078):<br>Short stature, chondrodysplasia with brachydactyly, joint dislocations, micrognathia, cleft palate and facial dysmorphism [38].<br><br>**- Catel-Manzke syndrome** (MIM - 616145):<br>Growth retardation, cleft palate with micrognathia, isolated knee hyperlaxity, abnormally shaped phalanges and carpal synostosis [39].
**Defects in UDP-sugar synthesis and transport**	*SLC35D1*<br><br>Transport of UDP-GlcA, UDP-GalNAc, UDP-GlcNAc, UDP-Gal, and UDP-Xyl in the ER.	**- Schneckenbecken dysplasia** (MIM - 269250):<br>Lethal chondrodysplasia, snail-like pelvis, flattened hypoplastic vertebral bodies, short ribs, short and wide fibulae, short and broad long bones with a dumbbell-like appearance, and precocious ossification of the tarsus [40].
	SLC35A3<br><br>UDP-GlcNAc transport in the Golgi appararus	**- SLC35A3-CDG - Autism spectrum disorder-epilepsy-arthrogryposis syndrome** (MIM - 615553):<br>Arthrogryposis, retromicrognathia, hypotonia, delayed psychomotor development, autism, seizures, microcephaly, and intellectual disability [41].
	SLC35A2<br><br>UDP-Gal transport in the Golgi apparatus	**- SLC35A2-CDG** (MIM - 3000896):<br>Epilepsy, delayed psychomotor development, hypotonia, skeletal abnormalities, facial dysmorphism, inverted nipples, vision and hearing abnormalities, cardiac diseases [42].

	CANT1<br>(Calcium activated nucleotidase)<br>ER/Golgi protein involved in the hydrolysis of the UDP released after the glycosylation reactions	**- Desbuquois dysplasia type 1** (MIM - 613165):<br>Severe growth retardation, joint laxity, short extremities, scoliosis, short long bones with metaphyseal splay, 'Swedish key' appearance of the proximal femur and advanced carpal and tarsal ossification [43].
	TGDS<br>Unknown function (possible involvement in UDP-sugar biosynthesis)	**- Catel-Manzke syndrome** (MIM:616145):<br>Pierre robin sequence (cleft palate, glossoptosis, micrognatia, breathing difficulties), hyperphalangy, joint hypermobility, short long bones [44].
	UGDH<br>Synthesis of UDP-GlcA from UDP-glucose	**- Epileptic encephalopathy with developmental delay (MIM – 603370/618792)**<br>Severe developmental delay, epileptic encephalopathy, facial dysmorphisms [45].
**Defects linked to impaired Golgi homeostasis**	*TMEM165*<br>Golgi localized Ca^{2+} - Mn^{2+} / H^{+} antiporter	**- TMEM165-CDG** (MIM:614727):<br>Psychomotor and growth retardation, facial dysmorphism, skeletal dysplasia with osteoporosis, scoliosis [46].
	COG4<br>(Conserved oligomeric Golgi - subunit 4)<br>Tethering complex involved in the retrograde trafficking from the Golgi apparatus to the ER	**- Saul Wilson syndrome** (MIM:606976):<br>Short stature, characteristic craniofacial dysmorphisms, prominent forehead, cataract, microencephaly, clubfoot, brachydactyly, irregularities of the vertebral bodies [47].

SLC10A7<br>Ca^{2+} influx regulation across intracellular membranes	**- Skeletal dysplasia and amelogenesis imperfecta** (unreferenced):<br>Short stature, microretrognathia, dislocations, monkey wrench appearance of the femora, short long bones, advanced carpal and tarsal ossification, severe tooth abnormalities [48].
GORAB (Golgin Rab6-interacting protein)<br>Trans-Golgi protein involved in retrograde trafficking	**- Gerodermia osteodysplastica** (MIM:231070):<br>Lax and wrinkled skin, osteoporosis with spontaneous fractures, short stature, joint dislocations, and intellectual disabilities [49].<br><br>**-**

References

1. Kolset, S. O.; Tveit, H. Serglycin – Structure and biology. *Cell **2008**. Mol. Life Sci. vol. 65, p. 1073–1085.*
2. Alexopoulou, A.; Multhaupt N.; Couchman, H. A. Syndecans in wound healing, inflammation and vascular biology. *The International Journal of Biochemistry & Cell Biology* **2007**, *vol. 39, p. 505–528.*
3. Song, H. H.; Filmus, J. The role of glypicans in mammalian development. *Biochimica et Biophysica Acta* - General Subjects ***2002**, vol. 1573, p. 241–246.*
4. Kiani, C.; Chen, L.; Wu, Y. J.; Yee, A. J.; Yang, B. B. Structure and function of aggrecan. *Cell Research **2002**, vol. 12, p. 19–32.*
5. Jan, A. T.; Lee, E. J.; Choi, I. Fibromodulin: A regulatory molecule maintaining cellular architecture for normal cellular function. *The International Journal of Biochemistry & Cell Biology **2016**, vol. 80, p. 66–70.*
6. Rühland, C. et al. The glycosaminoglycan chain of decorin plays an important role in collagen fibril formation at the early stages of fibrillogenesis. *FEBS J* **2007**, *vol. 274, p. 4246–4255.*
7. Csordás, G. et al. Sustained down-regulation of the epidermal growth factor receptor by decorin. A mechanism for controlling tumor growth in vivo. *J Biol Chem **2000**, vol. 275, p. 32879–32887.*
8. Kram, V. et al. Biglycan in the Skeleton. *J Histochem Cytochem **2020**, vol. 68, p. 747–762.*
9. Ye, Y. et al. Glycosaminoglycan chains of biglycan promote bone morphogenetic protein-4-induced osteoblast differentiation. *Int J Mol Med **2012**, vol. 30, p.1075–1080.*
10. Seppinen, L.; Pihlajaniemi, T. The multiple functions of collagen XVIII in development and disease. *Matrix Biology **2011**, vol. 30, p. 83–92.*
11. Kali, A.; Shetty, K. S. R. Endocan: A novel circulating proteoglycan. *Indian J Pharmacol **2014**, 46, 579–583.*
12. Lord, M. S., Melrose, J., Day, A. J. & Whitelock, J. M. The Inter-α-Trypsin Inhibitor Family: Versatile Molecules in Biology and Pathology. *J Histochem Cytochem **2020**, vol. 68, p. 907–927.*
13. Partenheimer, A.; Schwarz, K.; Wrocklage, C.; Kölsch, E.; Kresse, H. Proteoglycan form of colony-stimulating factor-1 (proteoglycan-100). Stimulation of activity by glycosaminoglycan removal and proteolytic processing. *The Journal of Immunology **1995,** vol. 155, p. 5557–5565.*
14. Chang, M. Y., Olin, K. L., Tsoi, C., Wight, T. N. & Chait, A. Human monocyte-derived macrophages secrete two forms of proteoglycan-macrophage colony-stimulating factor that differ in their ability to bind low density lipoproteins. *J Biol Chem **1998**, vol. 273, p. 15985–15992.*
15. Lamant, M. et al. ApoO, a novel apolipoprotein, is an original glycoprotein up-regulated by diabetes in human heart. *J Biol Chem **2006**, vol. 281, p. 36289–36302.*
16. Weijler, A. M.; Schmidinger, B.; Kapiotis, S.; Laggner, H. & Hermann, M. Oleic acid induces the novel apolipoprotein O and reduces mitochondrial membrane potential in chicken and human hepatoma cells. *Biochimie **2018**, vol. 147, p. 136–142.*
17. Bui, C. et al. XYLT1 Mutations in Desbuquois Dysplasia Type 2. The American Journal of Human Genetics ***2014**, vol. 94, p. 405–414.*
18. Munns, C. F et al. Homozygosity for frameshift mutations in XYLT2 result in a spondylo-ocular syndrome with bone fragility, cataracts, and hearing defects. *Am. J. Hum. Genet. **2015**, vol. 96, p. 971-978.*
19. Almeida, R et al. Cloning and expression of a proteoglycan UDP-galactose:beta-xylose beta1,4-galactosyltransferase I. A seventh member of the human beta4-galactosyltransferase gene family. *J. Biol. Chem. **1999**, vol. 274, p. 26165-26171*
20. Malfait, F. et al. Defective Initiation of Glycosaminoglycan Synthesis due to B3GALT6 Mutations Causes a Pleiotropic Ehlers-Danlos-Syndrome-like Connective Tissue Disorder. *Am J Hum Genet **2013**, vol. 92, p. 935–945.*
21. Baasanjav, S et al. Faulty Initiation of Proteoglycan Synthesis Causes Cardiac and Joint Defects. Am J Hum Genet ***2011**, vol. 89, p. 15-27.*
22. Kuroda, Y. et al. A novel gene (FAM20B encoding glycosaminoglycan xylosylkinase) for neonatal short limb dysplasia resembling Desbuquois dysplasia. *Clinical Genetics **2019**, vol. 95, p. 713–717.*
23. Vodopiutz, J et al. Chondroitin Sulfate N-acetylgalactosaminyltransferase-1 (CSGalNAcT-1) Deficiency Results in a Mild Skeletal Dysplasia and Joint Laxity. *Human Mutation **2017**, vol. 38, no 1, p. 34-38.*
24. Li, Y. et al. Temtamy preaxial brachydactyly syndrome is caused by loss-of-function mutations in chondroitin synthase 1, a potential target of BMP signaling. *Am. J. Hum. Genet. **2010**, vol. 87, p. 757–767.*
25. Volpi, S. et al. EXTL3 mutations cause skeletal dysplasia, immune deficiency, and developmental delay. *J Exp Med **2017**, vol. 214, p. 623–637.*
26. Ahn, J *et al.* Cloning of the putative tumour suppressor gene for hereditary multiple exostoses (EXT1). *Nat Genet **1995**, vol. 11, p. 137-143.*

27. Stickens, D *et al.* The EXT2 multiple exostoses gene defines a family of putative tumour suppressor genes. *Nat Genet **1996**, vol. 14, p. 25-32.*
28. Müller, T. *et al.* Loss of dermatan sulfate epimerase (DSE) function results in musculocontractural Ehlers–Danlos syndrome. *Hum Mol Genet **2013**, vol. 22, p. 3761–3772.*
29. Hästbacka, J *et al.* The diastrophic dysplasia gene encodes a novel sulfate transporter: positional cloning by fine-structure linkage disequilibrium mapping. *Cell **1994**, vol. 78, p. 1073-1087.*
30. Thiele, H *et al.* Loss of chondroitin 6-O-sulfotransferase-1 function results in severe human chondrodysplasia with progressive spinal involvement. *PNAS **2004**, vol. 101, p. 10155-10160.*
31. Akama, T. O *et al.* Macular corneal dystrophy type I and type II are caused by distinct mutations in a new sulphotransferase gene. *Nat Genet **2000**, vol. 26, p. 237-241.*
32. Guo, Y. *et al.* Decreased Expression of CHST-12, CHST-13, and UST in the Proximal Interphalangeal Joint Cartilage of School-Age Children with Kashin-Beck Disease: an Endemic Osteoarthritis in China Caused by Selenium Deficiency. *Biol Trace Elem Res **2019**, vol. 191, p. 276–285.*
33. Dündar, M *et al.* Loss of dermatan-4-sulfotransferase 1 function results in adducted thumb-clubfoot syndrome. *Am. J. Hum. Genet. **2009**, vol. 85, p. 873-882.*
34. Miyake, N *et al.* Loss-of-function mutations of CHST14 in a new type of Ehlers-Danlos syndrome. *Human Mutation **2010**, vol. 31, p. 966-974, 2010.*
35. ul Haque, M. F *et al.* Mutations in orthologous genes in human spondyloepimetaphyseal dysplasia and the brachymorphic mouse. *Nature Genetics **1998**, vol. 20, Art. n° 2.*
36. Miyake, N. *et al.* PAPSS2 mutations cause autosomal recessive brachyolmia. *J Med Genet **2012**, vol. 49, p. 533–538.*
37. Oostdijk, W. *et al.* PAPSS2 deficiency causes androgen excess via impaired DHEA sulfation--in vitro and in vivo studies in a family harboring two novel PAPSS2 mutations. *J. Clin. Endocrinol. Metab. **2015**, vol. 100, E672-680.*
38. Vissers, L. E. L. M. *et al.* Chondrodysplasia and Abnormal Joint Development Associated with Mutations in IMPAD1, Encoding the Golgi-Resident Nucleotide Phosphatase, gPAPP. *Am J Hum Genet **2011**, vol. 88, p. 608–615.*
39. Nizon, M. *et al.* IMPAD1 mutations in two Catel-Manzke like patients. *American Journal of Medical Genetics Part A **2012,** 158A, 2183–2187.*
40. Furuichi, T. *et al.* Identification of loss-of-function mutations of SLC35D1 in patients with Schneckenbecken dysplasia, but not with other severe spondylodysplastic dysplasias group diseases. *J. Med. Genet. **2009**, vol. 46, p. 562–568.*
41. Edvardson, S. *et al.* Mutations in SLC35A3 cause autism spectrum disorder, epilepsy and arthrogryposis. *J. Med. Genet. **2013,** vol. 50, p. 733–739.*
42. Ng, B. G. *et al.* Mosaicism of the UDP-Galactose Transporter SLC35A2 Causes a Congenital Disorder of Glycosylation. *Am J Hum Genet **2013**, vol. 92, 632–636.*
43. Huber, C. *et al.* Identification of CANT1 Mutations in Desbuquois Dysplasia. *American Journal of Human Genetics **2009**, 85, vol. 706.*
44. Boschann, F. *et al.* TGDS pathogenic variants cause Catel-Manzke syndrome without hyperphalangy. *Am J Med Genet A **2020**, vol. 182, p. 431–436.*
45. Hengel, H. *et al.* Loss-of-function mutations in UDP-Glucose 6-Dehydrogenase cause recessive developmental epileptic encephalopathy. *Nat Commun **2020**, vol. 11, p. 595.*
46. Foulquier, F. *et al.* TMEM165 Deficiency Causes a Congenital Disorder of Glycosylation. *Am J Hum Genet **2012**, vol. 91, p. 15–26.*
47. Ferreira, C. R. *et al.* A Recurrent De Novo Heterozygous COG4 Substitution Leads to Saul-Wilson Syndrome, Disrupted Vesicular Trafficking, and Altered Proteoglycan Glycosylation. *Am J Hum Genet **2018**, vol.* 103, p. 553–567.
48. Laugel-Haushalter, V. *et al.* A New SLC10A7 Homozygous Missense Mutation Responsible for a Milder Phenotype of Skeletal Dysplasia With Amelogenesis Imperfecta. *Front Genet 2019, vol.* 10, p. 504.
49. Rajab, A. *et al.* Geroderma osteodysplasticum hereditaria and wrinkly skin syndrome in 22 patients from Oman. *Am. J. Med. Genet. A **2008**, vol. 146A, p. 965–976.*
